# Hindlimb Ischemia Impairs Endothelial Recovery and Increases Neointimal Proliferation in the Carotid Artery

**DOI:** 10.1038/s41598-017-19136-6

**Published:** 2018-01-15

**Authors:** Sabato Sorrentino, Claudio Iaconetti, Salvatore De Rosa, Alberto Polimeni, Jolanda Sabatino, Clarice Gareri, Francesco Passafaro, Teresa Mancuso, Laura Tammè, Chiara Mignogna, Caterina Camastra, Giovanni Esposito, Antonio Curcio, Daniele Torella, Ciro Indolfi

**Affiliations:** 1 Division of Cardiology, Department of Medical and Surgical Sciences, Magna Graecia University, Catanzaro, Italy; 2 Department of Medicine, Duke University, Durham, 27710 NC USA; 3 Department of Health Science, University “Magna Graecia”, 88100 Catanzaro, Italy; 4 Division of Cardiology, Department of Advanced Biomedical Sciences, University of Naples “Federico II”, Naples, Italy; 5 URT-CNR of IFC, Magna Graecia University, Catanzaro, Italy

## Abstract

Peripheral ischemia is associated with higher degree of endothelial dysfunction and a worse prognosis after percutaneous coronary interventions (PCI). However, the role of peripheral ischemia on vascular remodeling in remote districts remains poorly understood. Here we show that the presence of hindlimb ischemia significantly enhances neointima formation and impairs endothelial recovery in balloon-injured carotid arteries. Endothelial-derived microRNAs are involved in the modulation of these processes. Indeed, endothelial miR-16 is remarkably upregulated after vascular injury in the presences of hindlimb ischemia and exerts a negative effect on endothelial repair through the inhibition of RhoGDIα and nitric oxide (NO) production. We showed that the repression of RhoGDIα by means of miR-16 induces RhoA, with consequent reduction of NO bioavailability. Thus, hindlimb ischemia affects negative carotid remodeling increasing neointima formation after injury, while systemic antagonizzation of miR-16 is able to prevent these negative effects.

## Introduction

Peripheral Arterial Disease (PAD) affects approximately 20% of adults over the age of 50, exposing them to the negative impact of peripheral ischemia^1–3^. However, half of these subjects are asymptomatic. Hence, the actual impact of peripheral ischemia is largely under-estimated, leading to under-treatment and seriously undermining prevention strategies^4,5^. Interestingly, in patients with multidistrectual atherosclerosis the presence of peripheral ischemia is associated to a further increase in mortality independently of classical cardiovascular risk factors^6,7^. In line with these observations, it was shown that limb ischemia might remotely influence atherosclerotic vascular remodeling in coronary and cerebral districts^8,9^. Indeed, the presence of limb ischemia is often associated to endothelial dysfunction^10^, and consequently to a more severe atherosclerosis of remote districts^11^. Interestingly, patients with PAD and peripheral ischemia undergoing percutaneous coronary intervention (PCI) have a significantly poorer prognosis suggesting a remote negative impact of peripheral ischemia on coronary artery disease^12,13^. This picture has not improved with the recent significant progresses in medical therapy and introduction of the latest generation of drug eluting stents (DES)^14^. Indeed, it is known that limb ischemia can negatively influence vascular remodeling in other districts, such as coronary and cerebral arteries^12–15^.

MicroRNA (miRNAs), small noncoding RNAs, have emerged in the last decade as epigenetic regulators of essential biological processes^15–17^. In particular, they play a key role in the pathophysiology of atherosclerosis, including restenosis after PCI^18–20^, and could be used as circulating biomarkers^21–24^. Recent studies showed that miRNAs could modulate both vascular smooth muscle cells (VSMC) and the endothelium in response to vascular injury^25^. Moreover, miRNAs can mediate intercellular crosstalk, involving different cells of the vessel wall.

However, despite it has been described that peripheral ischemia can negatively influence remote vascular remodeling^12–15^, at the present time it is currently unclear how peripheral ischemia may influence vascular response to injury in a remote district, and whether miRNAs are involved in these phenomena. In this context, aim of the present study was to assess the remote effects of hindlimb ischemia on neointimal proliferation and endothelial recovery after balloon injury of the carotid artery, to investigate the molecular mechanisms underlying these phenomena.

## Results

### Effects of hindlimb ischemia on neointimal formation and endothelial recovery after carotid injury

**Figure 1:**
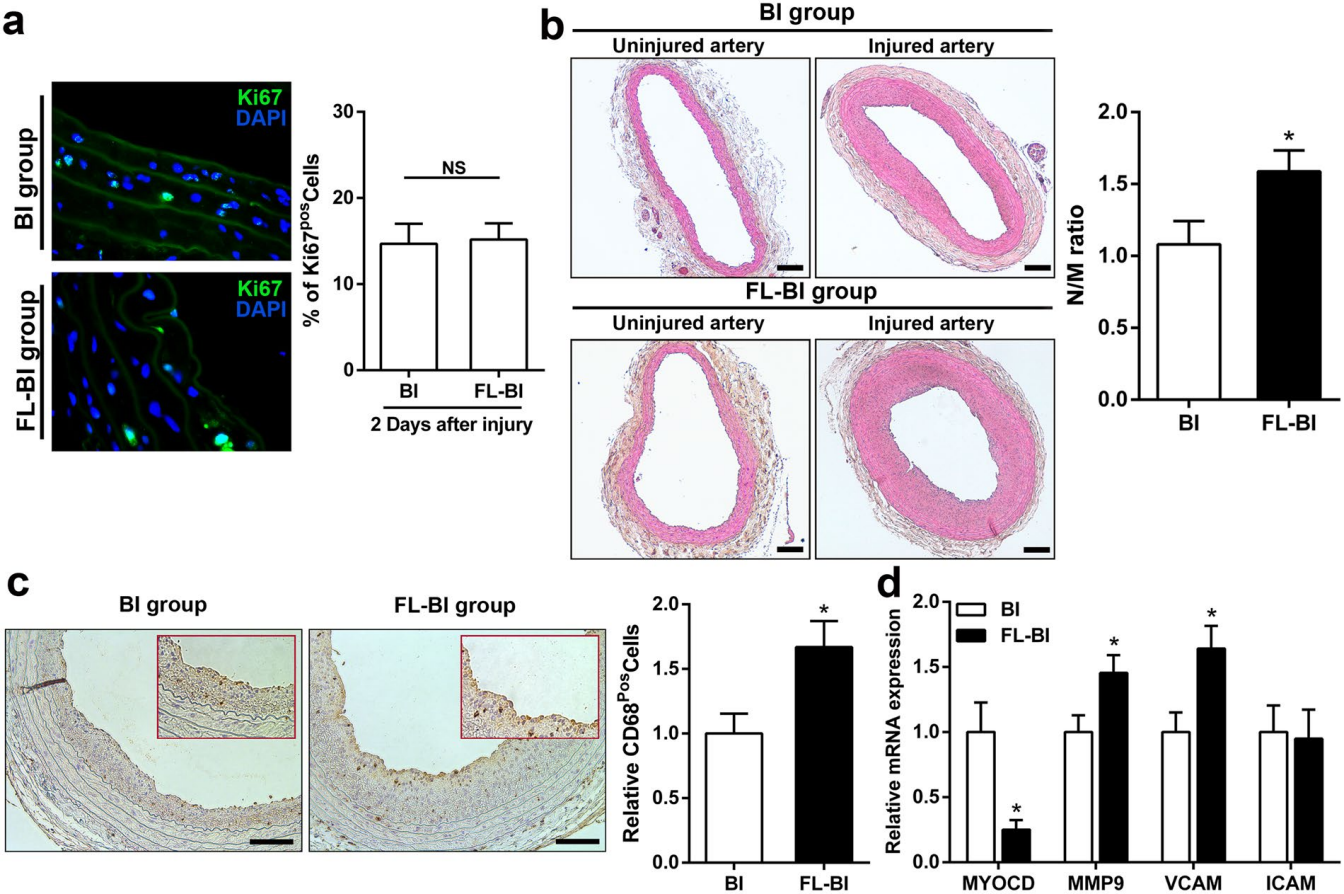
Effects of hindlimb ischemia on vascular carotid remodeling. (**a**) Left: Representative images of ki67 staining (green) in balloon-injured carotid arteries at 2 days after injury. Nuclei were stained with 4′,6-diamidino-2-phenylindole (DAPI). Right: Quantitative data derived from arterial sections stained with Ki67. Carotid arteries were explanted from BI and FL-BI groups at 2 days after balloon injury. NS: not significant. (**b**) Left: Representative images of haematoxylin and eosin staining in balloon-injured carotid arteries at 14 days in BI and FL-BI groups. Scale bars, 100 µm. Right: Bar graphs represent the morphometric analysis of arterial sections. Neointima /media ratio of arteries in differently treated groups is shown. Quantitative data derived from arterial sections at different levels from each animal in each group. *P < 0.05 versus BI group; n = 10 for group. (**c**) Left: Representative sections of carotid arteries stained for CD68^+^ macrophages (brown). Carotid arteries were explanted from BI and FL-BI groups at 14 days after balloon injury. Right: Quantitative analysis from arterial sections stained with CD68^+^cells. *P < 0.05 versus BI group; n = 7. (**d**) Relative expression of MYOCD, MMP9, VCAM and ICAM mRNA transcripts in balloon-injured arteries, evaluated at 14 days after balloon injury. *P < 0.01 versus BI group; n = 7.

To evaluate the effects of limb ischemia on vascular response to injury in a remote district, we performed a carotid artery balloon injury model in rats previously treated with unilateral ligation of the femoral artery (complete study design in Supplemental Fig. 1). The blood flow was evaluated by laser doppler imaging at day 0, 7, 14 and 28 after hindlimb ischemia to assess the efficiency of the ligation procedure and the development of collateral blood flow (Supplemental Fig. 2A). We first evaluated the effect of hindlimb ischemia on vascular remodeling at 2 days after injury. At this time point, no substantial difference in the expression levels of the specific VSMC markers SM22 and Calponin (Supplemental Fig. 2B) or proliferation (Fig. 1a and Supplemental Fig. 2C) in injured arteries was found between BI and FL-BI groups. Furthermore, no significant difference was observed at 5 days after injury in the expression levels of the differentiation markers ACT2 and SM-MHC, as well as the migration markers MMP9 and MMP2 between the BI and FL + BI groups (Supplemental Fig. 3).

However, the neointimal area was significantly larger in rats with hindlimb ischemia (neointima/media ratio = 1,58 ± 0,192 versus 1,18 ± 0,138 of control group, n = 10) at 14 days (Fig. 1b).

Macrophage infiltration reflects the degree of vascular remodeling after balloon injury^26^. Interestingly, staining of carotid sections, using CD68 specific antibody, showed a significant increase in infiltration of macrophage cells in injured artery from rats undergoing femoral artery ligation (FL-BI 1,67 ± 0.21versus BI 1.0 ± 0.15 fold; n = 7; P < 0.05) compared to control group (Fig. 1c). In addition, the expression level of myocardin, a key component of molecular network behind the modulation of VSMCs-phenotype switch, was markedly decreased (Fig. 1d); loss of myocardin is associated with phenotypic transition and inflammatory activation, one of the earliest stages of vascular disease^27^. Conversely, a significant increase in the levels of MMP-9 and VCAM (1,46 and 1,64 fold, respectively; p < 0,01) markers and active partakers of vascular remodeling was observed in the experimental group with hindlimb ischemia (Fig. 1d).

### Effects of hindlimb ischemia on carotid artery endothelium after injury

**Figure 2:**
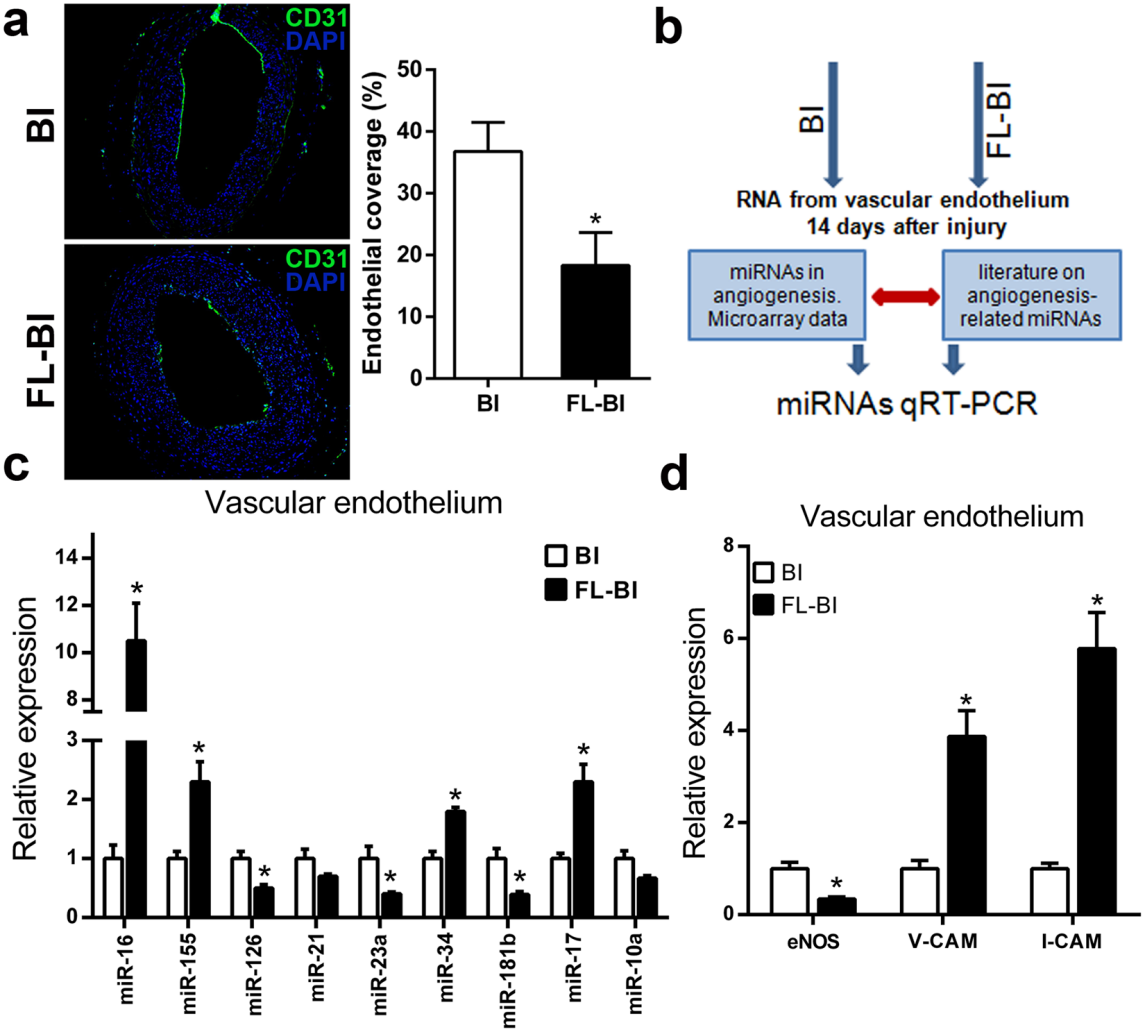
Effects of hindlimb ischemia on carotid artery endothelium. (**a**) Left: Representative sections of carotid arteries immunostained for the specific endothelial cell marker CD31. Nuclei were stained with 4′,6-diamidino-2-phenylindole (DAPI) in carotid arteries sections explanted from BI and FL-BI groups at 14 days after balloon injury. Scale bars = 50 µm. Right: Bar graphs represents the percentage of re-endothelializated circumference of the common carotid artery. *P < 0.05 versus BI group, n = 6 for group. (**b**) Schematic model of the experimental setup. (**c**) Expression levels of selected miRNAs in the carotid artery endothelium 14 days from injury. *P < 0.05 versus BI group; n = 6. (**d**) Relative expression of eNOS, VCAM and ICAM mRNA transcripts in carotid artery endothelium 14 days after injury. *P < 0.05 versus BI group; n = 6.

Several studies have shown an increase on neointima proliferation after angioplasty due to an inhibited endothelial recovery^28^. Since endothelial repair is an important component of the adaptive response of the vessel to injury, we examined this process in our model by immunofluorescent staining for CD31, a specific marker of endothelial cells. Compared to the control group, endothelial repair was significantly lower in rats undergoing femoral artery ligation, in terms of percentage of CD31-positive area (Fig. 2a). Hence, we hypothesized that the effect of hindlimb ischemia on endothelial repair in the vascular remote district might be an important mechanism related to exacerbate vessel response to injury. To test this possibility, we screened the expression of 9 miRNAs selected for their potential to modulate both angiogenesis and inflammatory processes (Fig. 2b and Supplemental Table). As shown in Fig. 2c, these miRNAs were differentially expressed in the group undergoing femoral ligation. In particular, 4 of them were upregulated and 3 downregulated. Among the others, miR-16 had the larger variation. In fact, the qRT-PCR analysis showed a 10-fold higher miR-16 levels in the endothelium from balloon-injured rat carotid arteries of animals underwent ligation of the femoral artery. Moreover, endothelial levels of miR-16 in FL + BI group are significantly up regulated at 21 through 28 days after carotid injury (Supplemental Fig. 4) showing a decline among these two time points, yet remaining higher when compared to BI group.

To further determine the effect of hindlimb ischemia on the endothelium of remote districts, we measured the expression level of key markers of endothelial activation as ICAM1 and VCAM1. As shown in Fig. 2d, intimal ICAM1 and VCAM1 mRNA levels were upregulated in FL-BI group (5,78 and 3,87 fold, respectively; n = 6; p < 0,01), compared to BI, suggesting inflammatory activation of endothelial cells (ECs). Interestingly, endothelial nitric oxide synthases (eNOS) expression was also significantly reduced (0,35 fold; n = 6; p < 0,01) in ECs from rats with chronic hindlimb ischemia (Fig. 2d). In order to evaluate the possible link between miR-16 expression and systemic inflammation, as related to hindlimb ischemia, we challenged Human Umbilical Vein Endothelial Cells (HUVEC) with pro-inflammatory cytokines known to play a key role in vascular inflammatory response. As shown in Supplemental Fig. 5A–C, no significant changes were observed in the expression of miR-16 in cells stimulated with tumor necrosis factor (TNFα), interleukin-8 (IL-8) or interleukin-1 β (IL-1 β). Of note, stimulation of HUVECs with interleukin-6 (IL-6) produced a significant and time-dependent increase of miR-16 levels (Supplemental Fig. 5D).

### Effect of miR-16 on endothelial cells

**Figure 3:**
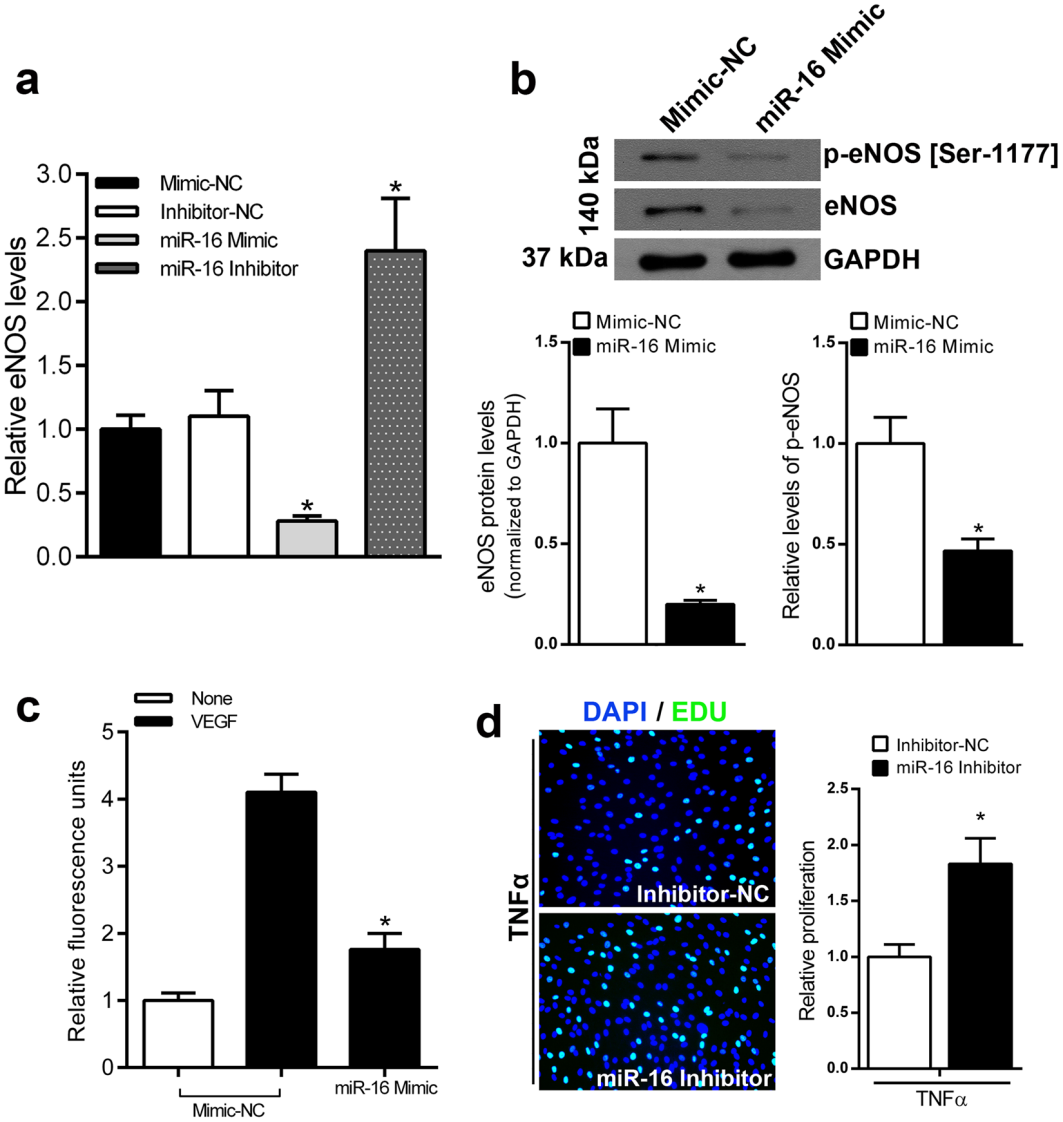
miR‐16 inhibits eNOS expression and activation (**a**) Cultured ECs were transfected with miR-16 Mimic, miR-16 Inhibitor, Mimic-NC, or Inhibitor-NC. The levels of eNOS mRNA were analyzed at 48 hours after transfection by real-time RT-PCR. *P < 0.01 versus cells transfected with Mimic-NC; n = 5. (**b**) eNOS expression and activation were determined using western blot analysis. Representative immunoblotting (upper) and quantification (lower) of eNOS and p-eNOS in HUVEC transfected with miR-16 Mimic. *P < 0,01 versus cells transfected with Mimic-NC; n = 4 (**c**) Measurement of Nitric Oxide (NO) production in ECs transfected with miR-16 mimic or mimic-NC. (*P < 0.05 versus cells transfected with mimic-NC). (**d**) Left: Representative images of EdU incorporation Assay in ECs in response to TNF-α. Right: Percentage of proliferating ECs (green) after transfection with miR-16 Inhibitor or Inhibitor-NC. *P < 0.05 vs. inhibitor NC.

It is known that miR-16 inhibits angiogenesis^29^. Given its high expression levels in ECs, we hypothesized that miR-16 mediate the impact of remote ischemia on endothelial recovery after injury of the carotid artery. Since we observed a reduced eNOS expression in ECs after injury, associated to a parallel increase in miR-16 levels, we evaluated whether eNOS was affected by miR-16 in our model. Hence, we measured eNOS expression levels in ECs treated with miR-16 Mimic, miR-16 Inhibitor or scramble negative control (NC). We found that overexpression of miR-16 reduce eNOS mRNA levels whereas the opposite effect was obtained with the *in vitro* inhibition of miR-16 (Fig. 3a). In line with these results, miR-16 overexpression was associated to a decreased eNOS protein expression and phosphorylation in ECs, as assessed by immunoblotting analysis (Fig. 3b). Furthermore, the DAF2/DA assay showed that inhibition of miR-16, result in a significant increase in nitric oxide (NO) production in response to VEGF (Fig. 3c).

We also found that peripheral ischemia was associated with an increase of macrophage infiltration after balloon injury, together with a pro-inflammatory activation of ECs. In order to evaluate the role of miR-16 in this phenomena, we tested the effect of miR-16 on TNF-α-induced EC proliferation. In the EDU incorporation assay, functional inhibition of miR-16 significantly increased the percentage of EDU-positive cells, indicating that inhibition of miR-16 attenuates the repressive effect of TNF-α on ECs proliferation (Fig. 3d).

These results demonstrate that the miR-16 suppresses eNOS expression and activity in vascular endothelial cells.

### miR-16 activates the RhoA pathway in EC

**Figure 4:**
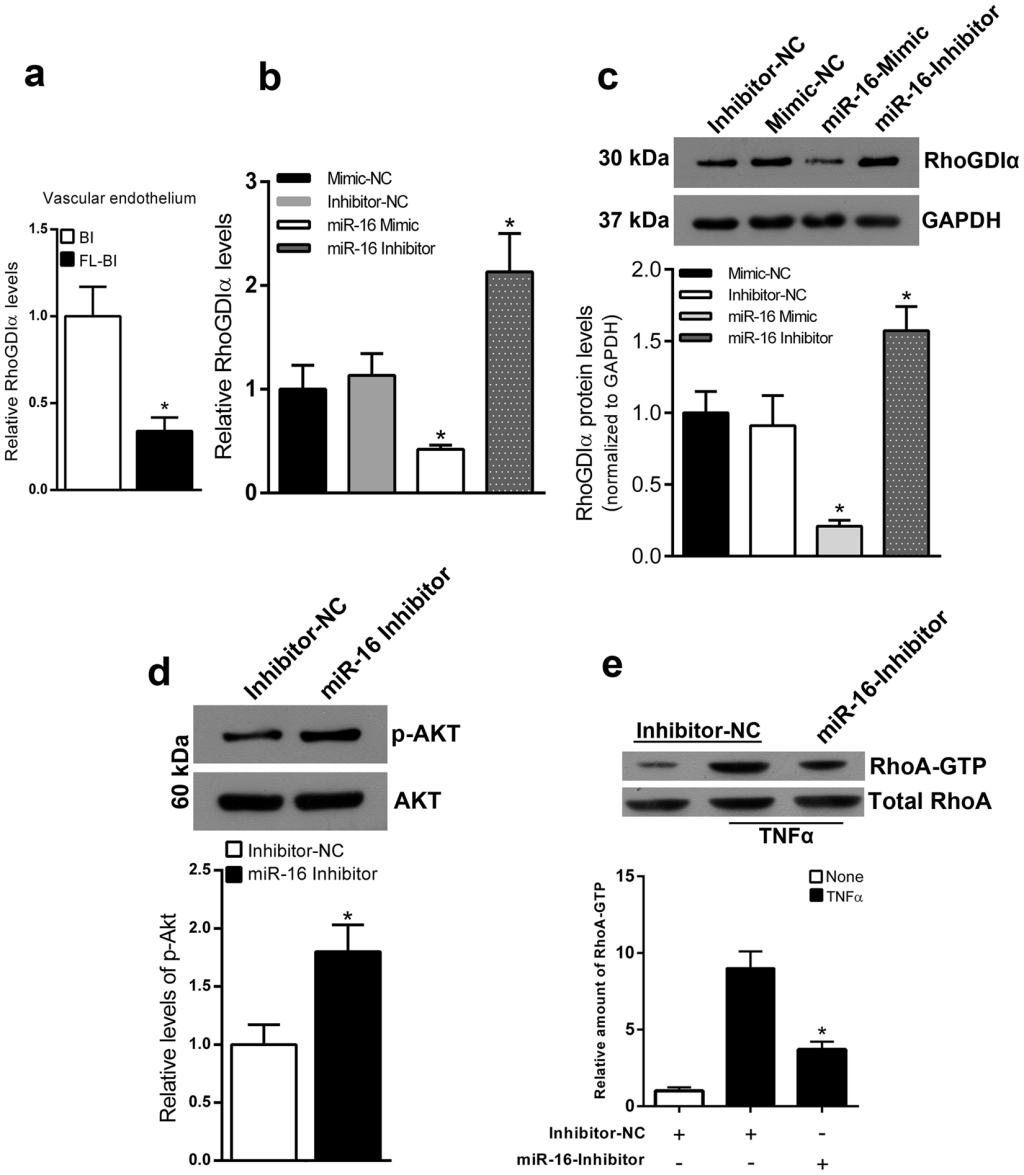
miR-16 upregulation leads to increased activation of RhoA by targeting RhoGDIα in endothelial cell. (**a**) Expression levels of RhoGDIα in ECs from rat carotid artery of BI and FL-BI groups at 14 days after balloon injury. *P < 0.01 versus control group; n = 5. (**b**) Cultured ECs were transfected with miR-16 Mimic, miR-16 Inhibitor, Mimic-NC, or Inhibitor-NC, showing modulation in RhoGDIα levels 48 hours after manipulation of miR-16 levels (real-time RT-PCR). *P < 0.05 versus cells transfected with Mimic-NC; n = 4. (**c**) Representative immunoblotting (upper) and quantification (lower) of RhoGDIα protein levels in HUVEC transfected with miR-16 Mimic, miR-16 Inhibitor, Mimic-NC or Inhibitor-NC. *P < 0,01 versus cells transfected with Mimic-NC; n = 4 (**d**) Representative immunoblotting (upper) and quantification (lower) of p-AKT in ECs transfected with miR-16 Inhibitor. *P < 0,01 versus cells transfected with Inhibitor-NC; n = 5. (**e**) Assay of RhoA activation in ECs transfected with miR-16 Inhibitor and stimulated with TNF. *P < 0.05 versus cells tranfected with Inhibitor-NC and treated with TNFα; n = 4.

Rho GDP dissociation inhibitor (RhoGDI) alpha, a member of the RhoGDI proteins, are an important regulators of the Rho family of small GTPases^30,31^. Interestingly, RhoGDIα has already been demonstrated as a direct target of miR-16^32^. We showed that overexpression of miR-16 inhibited the luciferase reporter activity confirming that RhoGDIα is a direct target of miR-16 (Supplemental Fig. 6). Interestingly, expression of RhoGDIα was significantly decreased in the endothelium of carotid artery derived from FL-BI group compared with the BI group (Fig. 4a).

We then examined the effect of miR-16 on RhoGDIα levels in ECs by using gain- and loss-of-function experiments. As shown in Fig. 4b,c, miR-16 overexpression and inhibition in endothelial cells were respectively responsible for down- and up-regulation of RhoGDIα mRNA and protein levels. Since small GTPase RhoA is known to play a crucial role in endothelial dysfunction, through down-regulation of eNOS and its phosphorylation^33,34^, we evaluated whether the functional inhibition of miR-16 inhibits its activity. As shown in Fig. 4d, the functional inhibition of miR-16 increased phosphorylation of AKT, decreasing the activation of the RhoA pathway.

Finally, ECs were exposed to TNFα for 3 h and activation of RhoA was measured using a pull-down assay. Of note, TNF-α treatment leads to marked RhoA activation in ECs (Fig. 4e). Importantly, on the contrary, the functional inhibition of miR-16 reduced the amount of RhoA-GTP approximately by 60%.

### Antagonizzation of miR-16 prevents the adverse impact exerted by hindlimb ischemia on remote arterial remodeling

**Figure 5:**
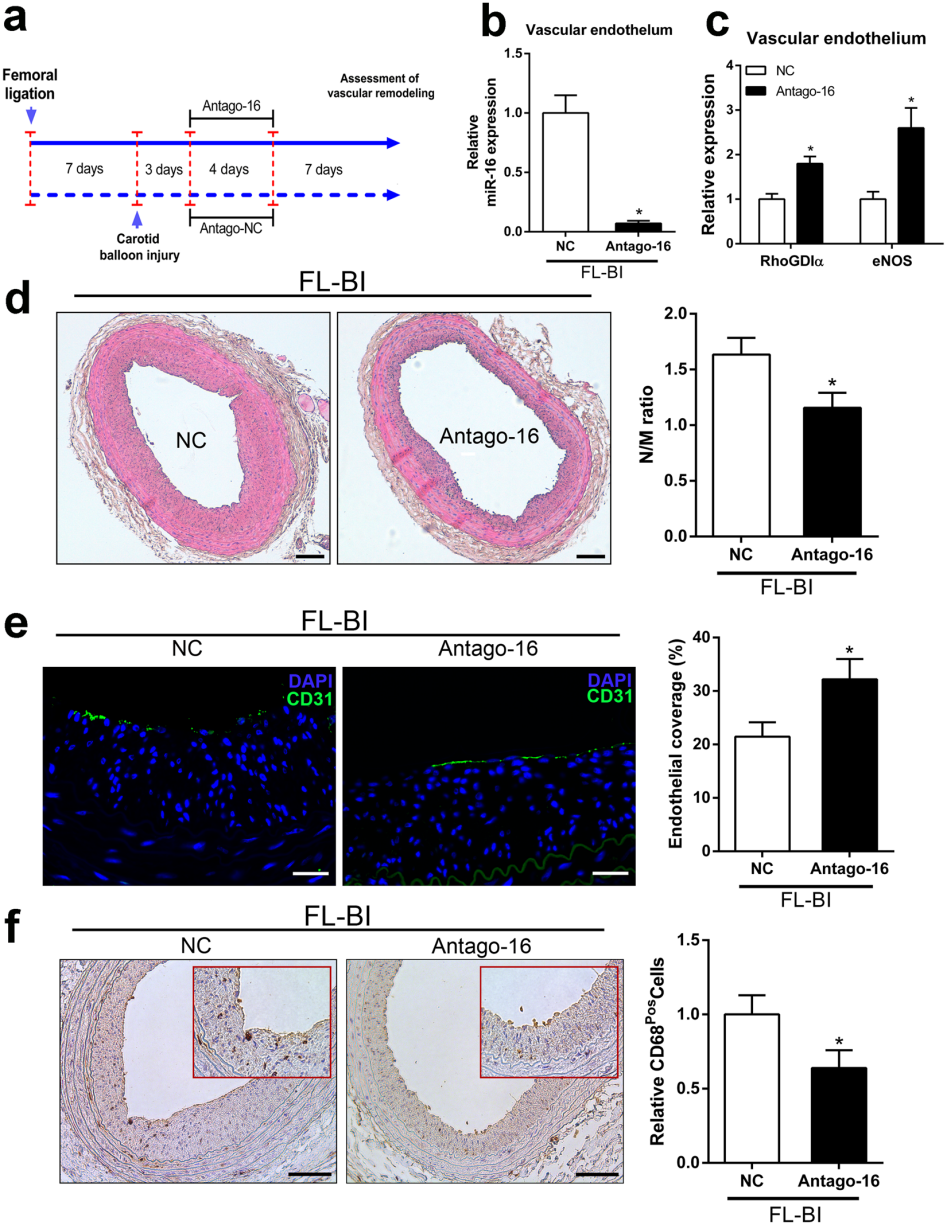
Systemic delivery of antagomiR-16 promotes endothelial recovery and inhibits neointima formation in the carotid artery of rats with hindlimb ischemia. (**a**) Schematic model of the experimental setup. (**b**) Expression levels of miR-16 in vascular endothelium. Total RNAs were obtained from vascular endothelium of rat carotid artery 14 days after injury. *P < 0.01 versus control group; n = 6. (**c**) Relative expression of RhoGDIα and eNOS mRNA transcripts in vascular endothelium of rat carotid artery 14 days after injury. *P < 0.05 versus control group; n = 6. (**d**) Left: Representative images of Haematoxylin and eosin staining in balloon-injured carotid arteries at 14 days in rats treated with or without Antago-16. Scale bars, 100 µm. Right: Bar graphs represent the morphometric analysis of arterial sections. Neointima /media ratio of arteries in differently treated groups is shown. *P < 0.05 versus NC group; n = 7. (**e**) Left: Representative sections of carotid arteries immunostained for the specific endothelial cell marker CD31. Nuclei were stained with 4′,6-diamidino-2-phenylindole (DAPI). Carotid arteries were explanted from experimental groups at 14 days after balloon injury. Scale bars = 50 µm. Right: Bar graphs represents the percentage of re-endothelializated circumference of the common carotid artery. *P < 0.05 versus rat treated with antagomir scrambled; n = 6 for group. (**f**) Left: Representative sections of carotid arteries stained for the macrophage (brown) marker CD68. Carotid arteries were explanted from rats at 14 days after balloon injury. Right: Quantitative data derived from arterial sections stained with CD68 positive cells. *P < 0.05 versus control; n = 5.

To confirm the involvement of miR-16 in mediating the remote effect of peripheral ischemia on vascular remodeling of the carotid artery after balloon injury, we performed systemic antagonism of miR-16 injecting a sequence-specific antagomir into the tail vein of the animals, as described in detail in Fig. 5a. As shown in Fig. 5b, miR-16 levels were efficiently reduced in vascular endothelium upon systemic delivery of antagomiR-16 (Antago-16). In parallel, changes in the expression levels of vascular RhoGDIα and eNOS were also observed (Fig. 5c). To evaluate the actual impact of miR-16 *in vivo*, we examined the role of miR-16 inhibition on neointima formation, endothelial repair and macrophage infiltration. Figure 5d shown representative hematoxylin and eosin-stained cross sections of right carotid arteries from antagomiR-scrambled and antagomiR-16-treated rats at 14 days after balloon injury. Treatment with antagomiR-16 resulted in a reduced neointima/media ratio in rats subjected to hindlimb ischemia (neointima/media ratio = 1,26 ± 0,14 versus 1,63 ± 0,162 of control group; n = 7). Furthermore, the treatment with Antago-miR-16 accelerated endothelial repair, as evident by the significant increase in the percentage area positive for the staining for CD31 (Fig. 5e).

Finally, staining of carotid sections with CD68 specific antibodies, to evaluate macrophage infiltration, showed a significant reduction in macrophage infiltration after miR-16 inhibition (Fig. 5f). Of note, concomitant overexpression of miR-16 and miR-15 was reported to inhibit neointimal hyperplasia in rats carotid artery after vascular injury^35^. To then evaluate the possibility that the systemic delivery of antagomir-16 affect switching of VSMCs from a contractile phenotype to a synthetic phenotype, expression levels of SMC marker genes were analyzed in injured artery of antagomir-16-treated rats previously subjected to unilateral ligation of the femoral artery. Expression analysis of ACTA2 and SM-MHC was then performed at different time points after vascular injury. As shown in Supplemental Fig. 7, no significant changes in the expression levels of VSMC markers were observed between control and antagomir-treated animals. These data indicate that miR-16 inhibition after injury does not significantly affect the VSMC phenotype and suggests that antagomir-16 could have an indirect effect on the reduction in neointimal formation by enhancing endothelial recovery after angioplasty.

## Discussion

The major findings of the present study are that: i) hindlimb ischemia remotely enhances neointimal hyperplasia and impairs endothelial recovery of the carotid artery after balloon injury; ii) miR-16 mediates, at least in part, the adverse impact of hindlimb ischemia on remote vascular remodeling at the carotid artery; iii) inhibition of miR-16, using a specific antagomir, is able to reduce the negative impact exerted by hindlimb ischemia.

The vascular response to injury is a process involving several cell types such as VSMCs, ECs and inflammatory cells^36,37^. It has been well established that VSMCs within adult animals can undergo profound and reversible changes in phenotype^38^, characterized by enhanced cellular proliferation, migration and decreased gene expression of contractile markers such as alpha-smooth muscle actin (ACTA2) and smooth muscle-myosin heavy chain (SM-MHC).

In the present manuscript, we showed for the first time that hindlimb ischemia leads to an impairment of the endothelial recovery and promotes an exaggerated intimal hyperplasia in balloon-injured carotid artery.

We hypothesized that the direct involvement of the endothelium in the modulation of neointima formation could explain the apparent gap between the virtual lack of impact on VSMC activation markers and the early effects exerted by hindlimb ischemia on neointima formation at 14 days. In fact, impairment of functional endothelial recovery can lead to multiple pathologic changes in the vascular walls^39,40^. To this regard we, and others, have previously shown an inverse relationship between endothelial integrity and neointimal proliferation^41,42^.

In the present study, we showed for the first time that several miRNAs are differentially expressed in the endothelial cells from balloon-injured carotid artery in rat with hindlimb ischemia. Remarkably, the most dramatic differential expression was observed for miR-16, known for its anti-angiogenic effect, exerted repressing fibroblast growth factor receptor-1 (FGFR1), Vascular endothelial growth factor (VEGF), and VEGF receptor-2 (VEGFR2)^29,43^. In fact, previous evidence found that increased miR-16 expression reduces proliferation and migration of ECs *in vitro* as well as the ability of these cells to form capillary-like structures *in vivo*^43^.

We also showed that miR-16 is a negative regulator of nitric oxide (NO) production in endothelial cells. In fact, miR-16 overexpression was able to reduce both the expression level of the endothelial nitric oxide synthase (eNOS) and its activity. Accordingly, we also observed that miR-16 overexpression reduces nitric oxide production in VEGF-stimulated ECs. These findings have a large potential impact, since the impairment of NO production is responsible for the development of a wide range of vascular diseases, such as atherosclerosis and peripheral vascular disease^31,44^. For example, it is well established that alterations in eNOS expression and activity may contribute to inhibit re-endothelialization and promote intimal hyperplasia after vascular injury^41,45^. In this context, the upregulation of miR-16 in the endothelium might impair endothelial recovery by repressing proliferation and migration as well as eNOS expression and activity in ECs.

We next identified Rho GDP dissociation inhibitor (RhoGDI) alpha as a downstream effector of miR-16 in ECs. RhoGDIα is a known target of miR-16 and published studies have shown the ability of this GDI to affect the activity of the Rho family of small GTPases including RhoA, CDC42, RAC1 and RAC2. RhoGDIα and members of the Rho family had already been associated to the development of cardiovascular diseases^30–33,46^.

We showed that miR-16 overexpression and inhibition were responsible respectively for the down- and up-regulation of RhoGDIα levels in ECs. Consistent with these findings, expression of RhoGDIα was significantly decreased in the endothelium of carotid arteries derived from rats subjected to femoral ligation. In that context, our results demonstrate that the overexpression of miR-16 induced by peripheral ischemia is responsible for the downregulation of RhoGDIα in ECs of remote vascular district, such as the carotid artery, accounting for the augmented degree of endothelial dysfunction after balloon injury of the carotid artery in animal with previous ligation of femoral artery. It has been well established that the small GTPase RhoA directly suppress NO production in the endothelium through reduction of both eNOS activity and gene expression. Studies have also shown that inhibition of RhoA leads to the activation of the Akt and eNOS in ECs^33^. Indeed, Akt can directly phosphorylate eNOS, leading to NO production. Our analysis revealed that functional inhibition of miR-16 led to increased phosphorylation of AKT on ECs. Furthermore, in the present study we showed that functional inhibition of miR-16 attenuates TNFα-induced RhoA activation. Altogether, these data strongly suggest that miR-16 indirectly modulates eNOS levels and function. Indeed, miR-16 directly targets for repression RhoGDIα that ensues in RhoA signaling pathway activation owing to eNOS down-regulation and functional inhibition.

The observation that the anti-angiogenic miR-16 was upregulated in the endothelium from rats with hindlimb ischemia suggests that miR-16 is at least in part responsible for the impairment of endothelial recovery in injured arteries. To test this hypothesis, an antisense oligo-nucleotide (Antago-miR) was used to knockdown miR-16 expression in endothelium of balloon-injured carotid artery. One of the major findings of the present study was that systemic administration of antagomirs allowed silencing of miR-16 in vascular endothelial cell, resulting in enhanced re-endothelialization, reduced neointima formation, and decreased vascular inflammation. We also demonstrated that *in vivo* inhibition of miR-16 significantly enhanced RhoGDIα and eNOS expression. These results further support our hypothesis that dysregulated miR-16 expression could mediate, at least in part, the observed effect of hind-limb ischemia on vascular response to injury. Of note, recent studies by Xu *et al*.^35^, using a balloon injury model, provided evidence that combined over-expression of miR-16 and miR-15b significantly inhibits neointima formation in injured carotid arteries. However, these results are only apparently in contrast with the findings described earlier, in which the authors evaluated the simultaneous over-expression of miR-15b and miR-16 on vascular remodeling but not the role of single over-expression or inhibition of miR-16. In this direction, our study shows the effect of a specific inhibition of miR-16 on the vascular remodeling, analyzing the endothelial recovery process. Additional studies are needed to evaluate the role of miR-16 on vascular response to injury and, in particular, on the role of VSMC. Indeed, no significant differences in Ki67 positive VSMCs between the two groups (FL-BI-to-BI Ki67 expression) were detected in the carotid artery media layer at 48 h after injury. However, this finding shows that hindlimb ischemia does not acutely exacerbate VSMC activation upon injury. Despite the latter, we show that hindlimb ischemia delays re-endothelialization overtime after injury. The latter most likely prolongs the activated state of VSMCs overtime,^47^ allowing for a few more rounds of their replication leading to an increased neointimal formation after injury in the hindlimb ischemia group. Furthermore, other mechanisms such as an increased homing of bone marrow-derived vascular progenitors^45^ and/or cell mobilization from the adventitia^48^ could have also contributed to the increased neointima formation upon injury by hindlimb ischemia. There are several limitations associated with this study. First, we have focused on RhoGDIα as target of miR-16, but we did not exclud that other targets of this miRNA might also contribute to the observed effects of chronic hindlimb ischemia on endothelial recovery and neointima formation. Second, it has been reported that PAD is associated with elevated plasma levels of several inflammatory markers^49^; however, we were not able to assess systemic levels of various pro-inflammatory cytokines in our experimental model. In response to pro-inflammatory cytokines, endothelial cells undergo rapid activation that contributes to several aspects of vascular disorders. In this study we show that Interleukin-6 (IL-6) induces the expression of miR-16 in endothelial cells *in vitro*. Interestingly, IL-6 is a multifunctional cytokine involved in several aspects of the atherogenic process, such as monocytes recruitment, endothelial cell activation, and inflammatory responses^50^. It is tempting to speculate that the induction of miR-16 in balloon-injured carotid artery endothelium of rats subjected to femoral ligation might be mediated, at least in part, by an increase of systemic inflammatory IL-6. Despite the results of these *in vitro* experiments, further *in vivo* studies are needed to demonstrate a direct link between IL-6 systemic levels and miR-16 vascular expression and function. A further limitation is represented by the experimental model. The acute occlusion of the femoral artery does not clearly reproduce the clinical setting of critical limb ischemia that usually develops over long time and includes several additional features that are not reproduced in the experimental animal model we employed. Hence, our results are mainly a proof of concept. Further studies are needed to evaluate whether our experimental hypothesis holds true in the clinical setting of chronic critical peripheral artery ischemia. On the other hand, the experimental model used in this study allowed us to prove the biological concept that remote ischemia has an impact on vascular remodeling in a central vascular district and to evaluate the underlying molecular mechanisms.

In conclusion, the present study provides a novel link between peripheral ischemia and vascular remodeling in a remote arterial district. In fact, we demonstrated from the first time that the presence of hindlimb ischemia significantly enhances neointima formation and impairs endothelial recovery in balloon-injured carotid arteries. We also found that *in vivo* inhibition of miR-16 significantly reduced these effects in the animal model. These results disclose a new regulatory mechanism, providing potentially targets and possible novel therapeutically strategies to beneficiary interfere with the adverse vascular remodeling in subjects with peripheral ischemia. Human studies should be performed to confirm the actual clinical impacts of these findings.

## Methods

### Animals

Animal procedures were performed conform to the directive 2010/63/EU of the European Parliament and approved by the Italian Ministry of Health and by Institutional Animal Care and Use Committee of Magna Graecia University. Male Wistar Rats, weighing 300–350 g, were randomly divided into two experimental groups: One group (Balloon Injury, BI) was subjected to carotid artery balloon injury, and the other group was subjected to unilateral femoral artery ligation (FL) and seven days after hindlimb ischemia subjected to balloon injury of the right common carotid artery (FL-BI). Rats from experimental groups, were anaesthetized by intraperitoneal injection of Zoletil (zolazepam hydrochloride and tiletamine hydrochloride; 20 mg/kg body weight) and Xylazine (10 mg/kg body weight). The rats were euthanized by an overdose of Zoletil (100 mg/kg body weight) and Xylazine (10%).

### Rat model of hindlimb ischemia

The surgical sites were shaved and treated with topical antiseptic. In order to establish a rat model of hind-limb ischemia, the left femoral arteries were isolated and completely occluded 5–6 mm distal to the inguinal ligament by ligation with 3–0 surgical silk through a small incision in inguinal area^51^. Blood perfusion of ischemic hindlimb was assessed using Laser Doppler perfusion images (PeriScan PIM3 system, Perimed, Sweden). LDPI index express ischemic to non-ischemic hind limb blood perfusion ratio.

### Rat carotid artery balloon injury model

Carotid artery balloon injury was performed in male Wistar rats as described and well validated in our previous studies. Using a dissecting microscope (Leica, 424), the right common carotid artery was exposed through a midline cervical incision. A 2 F Fogarty catheter (Baxter Edwards) was introduced via an arteriotomy of the external carotid artery into the common carotid artery. To produce an injury, the balloon was inflated at 1.5 atm and withdrawn three times through common carotid artery as previously described and validated in our laboratory^36,52,53^. The external carotid artery was then permanently ligated with a 5–0 silk suture, and the blood flow in the common carotid artery was restored.

### Morphometric analysis and immunohistochemistry of injured artery

Neointimal formation in balloon-injured arteries was evaluated 14 days after injury. Carotid arteries from experimental groups were embedded in paraffin and 5 µm cross sections were prepared. Morphometric analysis via computerized image analysis system (ImageJ v1.43) was performed in vessel sections stained with hematoxylin-eosin (H-E). The average of the nine sections was used as the value for one animal. The parameter for analysis of neointimal formation was neointimal to medial area ratio (N/M)^41,54^. Macrophage infiltration was evaluated on day 14 after injury using primary antibodies for CD68. In brief, the formalin-fixed/paraffin-embedded sections were deparaffinized, followed by epitope masking with Antigen Retroviral Reagent (R&D Systems, catalog number CTS016). The sections were then treated with Serum Blocking Reagent (R&D Systems, catalog number 865015), and incubated with CD68 antibody for overnight at 4 °C. The CD68 staining was assessed using a HRP-DAB cell staining kit (R&D Systems, Cell & Tissue Staining Kit) according to the protocol provided by the manufacturer. Sections were counterstained with hematoxylin, mounted, and photographed using a Leica microscope.

### Immunofluorescence staining

Endothelial repair in balloon-injured arteries was evaluated at 14 days after vascular injury using a primary antibody against the endothelial cell surface marker CD31 (R&D Systems, catalog #AF3628, 1:50 dilution). Briefly, paraffin-embedded injured arteries were sectioned (4 μm), and a goat anti-CD31/PECAM-1 primary antibody was incubated with the tissue sections at 4 °C overnight. Fluorescently labeled anti-goat secondary antibody was used for detection. Nuclei were stained with 4′,6-diamidino-2-phenylindole (DAPI) 1:1000 in PBS. Representative digital images were acquired with a 40 × objective using a Leica fluorescent microscope (Leica Microsystems, Wetzlar, Germany). The percentage of the peri-luminal perimeter that was positively stained with CD31 was determined using ImageJ analysis software (NIH, USA).

Proliferating VSMCs in balloon-injured arteries were identified using primary antibody (1:50 dilution) against the proliferation marker Ki67. At 2 days after vascular injury, the rats were sacrificed and the carotid arteries were harvested and processed as describe above. Several carotid sections of each group were then stained with DAPI. The number of Ki-67-positive nuclei within each vessel layer was counted by visual determination in five or more randomly fields per cross section. The amount of proliferating cells was calculated as the number of Ki67-positive cells/total cells^55^.

### Antagomir treatment

To evaluate the effects of miR-16 knockdown on vascular remodeling, we designed single stranded RNA, termed antagomir, complementary to specific sequence of miR-16 (Antago-16). Antago-16 and antagomir negative control (NC) molecule (Antago-NC), were synthesized and HPLC-purified by Fidelity Systems, Inc. The sequences for each oligo are as follows: Antago-16: 5′-GCCAAUAUUUACGUGCUGCUA-3′;Antago-NC: 5′-CAGUACUUUUGUGUAGUACAA-3′. Rats were subjected to hindlimb ischemia and seven days after femoral ligation subjected to balloon injury. Rats were then randomly divided into two groups, Antago-NC group and Antago-16 group. After balloon injury, solutions of Antag-16 (8 mg/kg) or Antago-NC (8 mg/kg) were infused systemically by tail vein at 3 and 7 days after injury. Animals were sacrificed at 14 days and the effects of miR-16 inhibition on neointimal formation, endothelial repair and vascular inflammation was evaluated using Hematoxylin/eosin, CD31, and CD68 staining respectively.

### Cell culture, transfection and reagents

Human Umbilical Vein Endothelial Cells (HUVEC, catalog number C-003–5C) were obtained from Life Technologies and were cultured in Endothelial Cell Growth Medium (EGM, Lonza) in a 5% CO2 humidified atmosphere at 37 °C. Cells between passages two and four were used in the experiments.

HUVECs were transfected using Lipofectamine RNAi MAX Transfection Reagent (Invitrogen) according to the manufacturer’s protocol. For gain of function studies, we used double-stranded RNAs (miR-16-Mimic, 10 nmol/L) that mimic endogenous miR-16 (Ambion). For down-regulation of miRNA activity, we used single stranded RNAs (miR-16-Inhibitor, 30nmol/L) designed to specifically inhibit endogenous miR-16 (Ambion). As negative controls, cells were transfected with the mirVana miRNA Mimic (Mimic-NC) or mirVana miRNA Inhibitor (Inhibitor-NC). HUVEC proliferation in response to TNFα was assessed using Click-it EdU Proliferation kit (Invitrogen) according to the manufacturer’s protocol. In brief, cells were cultured in 12-well plates at a density of 70.000 cells for well and transiently transfected with miR-16-Inhibitor (30nmol/L) or Inhibitor-NC (30nmol/L). Transfected HUVEC were serum starved by replacing with basal medium (EBM-2) for 18 hours. HUVEC were then stimulated with TNFα and incubated with 5-ethynyl-2′-deoxyuridine (EDU) at a concentration of 10µmol/L. After 18 hours, cells were fixed and stained to detect cell incorporation of the thymidine analogue, according to the manufacturer’s instructions. To investigate the effect of pro-inflammatory cytokine on miR-16 expression, HUVECs were incubated with IL-6 (I1395–10UG; Sigma Aldrich), IL-8 (I1645–10UG; Sigma Aldrich), IL-1β (I9401–5UG; Sigma Aldrich), and TNF-α (T6674–10UG; Sigma Aldrich) at the time points indicated.

### RNA extraction and Real-time PCR analysis

After explants of common carotid artery, the internal lumen of the vessels were perfused with 200 µl of Qiazol lysis reagent (Qiagen GmbH, Germany) and the endothelial-enriched RNA was extracted according to the protocol provided by the manufacturer. Total RNA samples were assessed for quantity and purity using a NanoDrop spectrophotometer (Thermo Scientific). In addition, RNA from vascular endothelium was tested for endothelial cell marker CD31 and VSMC-specific smooth muscle alpha-actin (ACTA2) by real time PCR (data no shown). Mature miRNAs expression was evaluated by real-time RT-PCR using TaqMan microRNA assays (Applied Biosystems). In brief, 15ng of total RNA was reverse-transcribed with stem–loop RT primer specific for miR-16, miR-155, miR-126, miR-21, miR-23a, miR-34, miR-181b, miR-17, miR-10a according to the manufacturer’s instructions. Real-time PCR was performed on the resulting cDNA using the complementary TaqMan primers. The expression of the housekeeping gene U6 was used as an endogenous control for the normalization of data.

TaqMan Gene Expression Assays (Applied Biosystems) was used for quantification of Myocardin (Assay ID:), MMP2 (Assay ID: Rn01538170_m1), CNN1 (Assay ID: Rn00582058_m1), c-Fos (Assay ID:) MMP9 (Assay ID: Rn00579162_m1), Rat ACTA2 (Assay ID: Rn01759928_g1), Rat MYH11 (Assay ID: Rn01530321_m1), Rat eNOS (Assay ID: Rn02132634_s1), Human eNOS (Assay ID: Hs01574665_m1), Rat VCAM (Assay ID: Rn00563627_m1), ICAM (Assay ID: Hs00164932_m1), Human VCAM (Assay ID: Hs01003372_m1), Rat ICAM (Assay ID: Rn00564227_m1), Human RhoGDIα (Assay ID: Hs00366348_g1) and Human GAPDH (Assay ID: Hs02758991_g1) transcripts.

In brief, total RNA was reverse-transcribed with High- Capacity cDNA Reverse Transcription Kits (Applied Biosystems). Real-time PCR was performed on the resulting cDNA using specific primers. The expression of the housekeeping gene GAPDH was used as an endogenous control for the normalization of data. The relative quantities of mRNA were determined using comparative ΔCT.

### Immunoblot analysis

Protein samples were isolated from HUVEC tranfected with miR-16-Mimic, miR-16-Inhibitor, Mimic-NC or Inhibitor-NC. Protein extracts obtained with NP40 Lysis Buffer (Thermo Fisher Scientific, catalog number #FNN0021) were separated on SDS–PAGE gels and western blot analyses were performed by standard protocols. The antibodies used were the following: polyclonal rabbit anti-eNOS antibody (Cell Signaling, catalog number #9572), goat polyclonal anti-phospho-eNOS (Ser 1177) antibody (Santa Cruz Biotech., sc-12972), AKT antibody (Cell Signaling, catalog number #9272), rabbit Phospho-Akt (Ser473) antibody (Cell Signaling, catalog number #193H12), mouse monoclonal RhoGDIα antibody (Santa Cruz Biotech., sc-373724) and rabbit polyclonal anti-GAPDH (FL-335) antibody (Santa Cruz Biotech., sc-25778). Quantification of Western blots was performed by densitometry using NIH ImageJ software. Full-length blots for the figures were presented in Supplemental Fig. 8.

### Nitric oxide assay

For determination of endothelial nitric oxide (NO) production, HUVEC were cultured in 12-well plates at a density of 100.000 cells for well and transiently transfected with miR-16 Mimic (30 nmol/L) or mimic-NC (30 nmol/L). After 36 hours, transfected HUVEC were seeded overnight in 96-well plates at a density of 40.000 cells per plate. Cells were then were incubated with or without VEGF (20ng/ml). Nitric oxide production from HUVEC in response to VEGF was then assessed using spectrofluorimeter Nitric Oxide Synthase Detection System, (Sigma-Aldrich, catalog number #FCANOS1) according to the manufacturer’s protocol. For fluorometric NO determination, the cell-permeable NO-sensitive 4,5-Diaminofluorescein diacetate (DAF-2DA) was used. Data expressed as relative fluorescence units (RFU) of NO concentration.

### *In vitro* RhoA Activation Assay

The activation of endogenous Rho GTPase family members RhoA was assessed using RhoA activation assay kit (Cell Biolabs, Inc., San Diego, CA). Briefly, the assay was performed on TNFα-stimulated ECs transfected with miR-16-Inhbitor or Inhibitor-NC. Transfected cells were lysed in buffer solution (125 mmol/LHEPES, 750 mMNaCl, 5% NP-40, 50 mM MgCl2, 5 mM EDTA, 10% Glycerol) provided with the kit. Lysates were then used for pull down assays according to manufactures instructions. RhoA expression was determined by a Western blot using mouse monoclonal anti-RhoA antibody (Cell Biolabs) and anti-mouse-horseradish peroxidase (HRP) conjugated secondary antibody (Santa Cruz Biotech). Quantification of Western blots was performed by densitometry using NIH ImageJ software.

### Luciferase Assay

Luciferase activity was measured by Dual-Luciferase Reporter Assay System (Promega, catalog number E1910) according to manufactures instructions. Cells were transfected using lipofectamine 3000 Transfection Reagent (Life Technologies, catalog number L3000008). In brief, 293 cells were co-transfected with miR-16-Mimic and firefly luciferase reporter constructs containing RhoGDIα3′UTR. After 48 hours, luciferase activity was then measured and normalized to the activity of the Renilla luciferase.

## Electronic supplementary material

Supplementary Dataset 1
