# Supplementary material for: Hindlimb Ischemia Impairs Endothelial Recovery and Increases Neointimal Proliferation in the Carotid Artery
Source: Sci Rep. 2018 Jan 15;8:761. doi: 10.1038/s41598-017-19136-6 (PMC5768880; doi:10.1038/s41598-017-19136-6)
Supplement: Supplementary file 1 — Supplementary Dataset 1 [file 41598_2017_19136_MOESM1_ESM.doc]

**SUPPLEMENTAL MATERIAL**

**Hindlimb Ischemia Impairs Endothelial Recovery and Increases Neointimal Proliferation in the Carotid Artery**

**Running title: Adverse Effects of Limb Ischemia on Damaged Artery**

Sabato Sorrentino^1^*;Claudio Iaconetti^1^*;Salvatore De Rosa^1^; Alberto Polimeni^1^; Jolanda Sabatino^1^; Clarice Gareri^2^; Francesco Passafaro^1^; Teresa Mancuso^1^; Laura Tammè^1^; Chiara Mignogna^3^; Caterina Camastra^3^; Giovanni Esposito ^4^; Antonio Curcio^1^; Daniele Torella^1^; Ciro Indolfi^1,5^

^1^Division of Cardiology, Department of Medical and Surgical Sciences, Magna Graecia University, Catanzaro, Italy.

^2^Department of Medicine, Duke University, Durham, NC

^3^Pathological Anatomy, Department of Health Science, University "Magna Graecia", 88100, Catanzaro, Italy.

^4^Division of Cardiology, Department of Advanced Biomedical Sciences, University of Naples "Federico II", Naples, Italy

^5^URT-CNR of IFC, Magna Graecia University, Catanzaro, Italy.

*both authors contributed equally to this work.

Corresponding author:

Ciro Indolfi, MD

Professor of Cardiology, Department of Medical and Surgical Sciences

Magna Graecia University Catanzaro 88100, Italy

E-mail: [indolfi@unicz.it](mailto:indolfi@unicz.it); Phone: +3909613647151; Fax: +3909613647153

**Supplemental Figure 1**

**
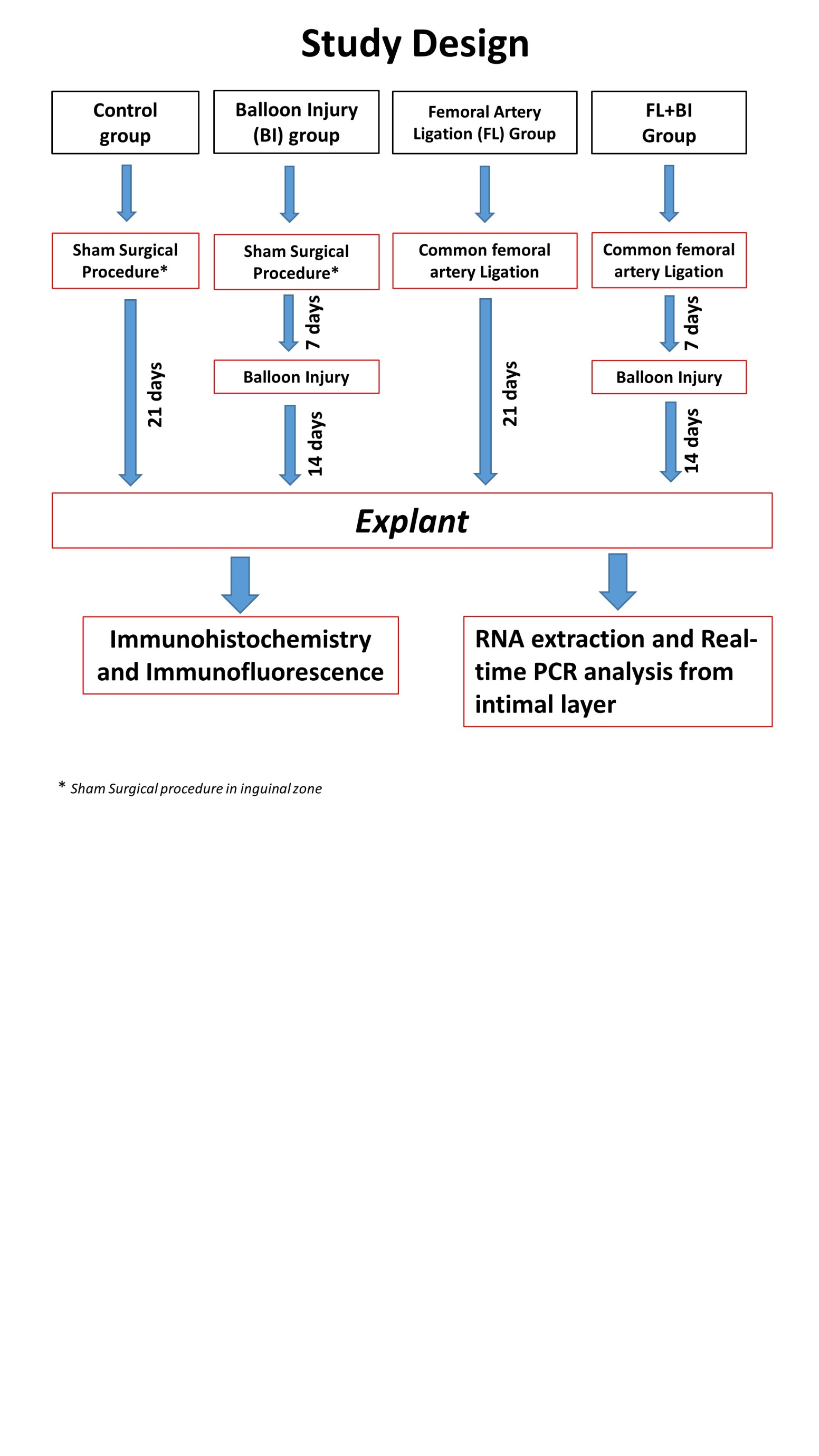
**

***Sham surgical procedure in left inguinal zone.**

**Supplemental Figure 2**

**
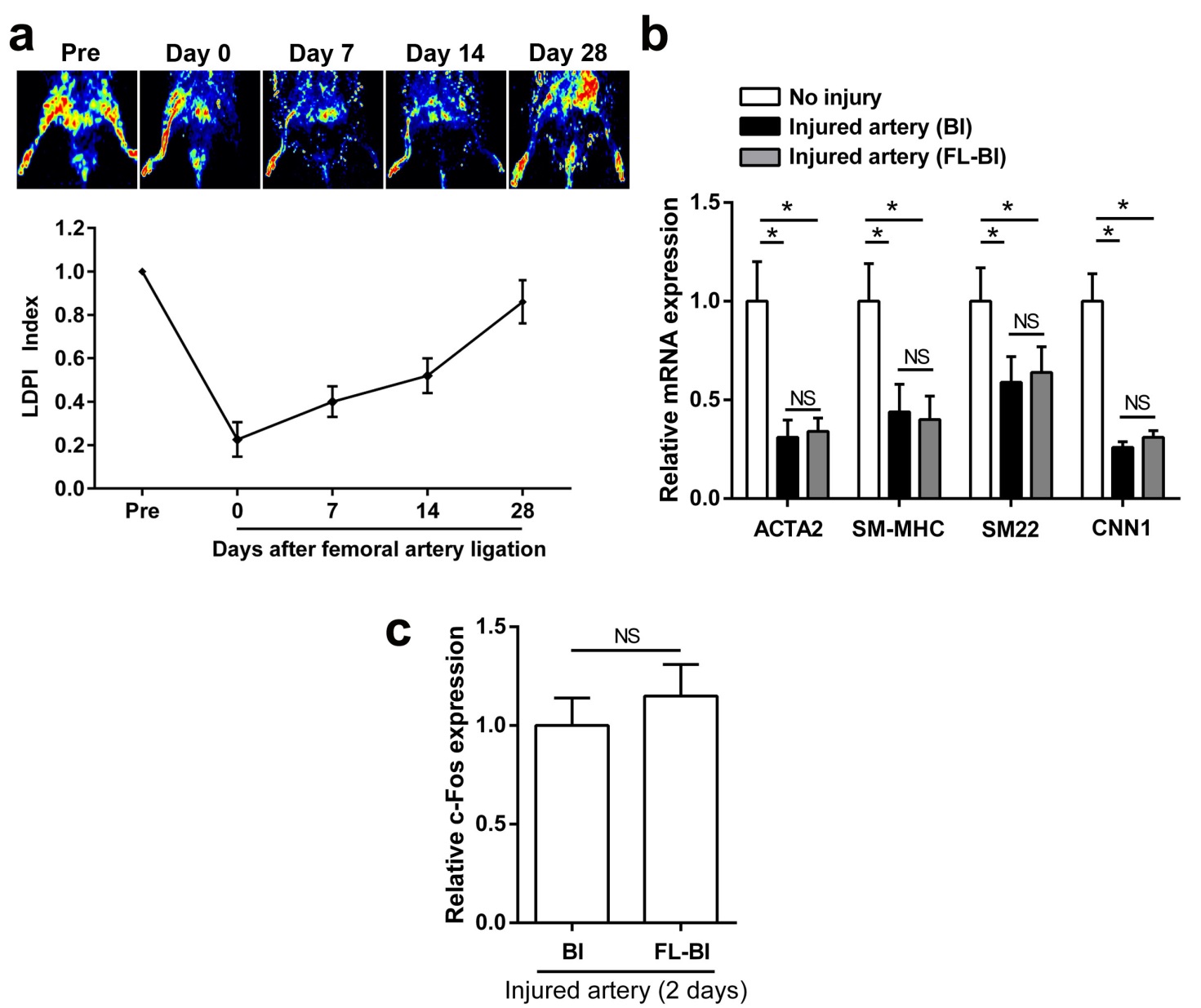
**

**Effects of critical hindlimb ischemia on vascular remodeling at 48 hours after balloon injury.**(a) Laser Doppler imaging and analysis with perfusion quantitation 0, 7, 14 and 28 days after surgery (n = 8) (b) Expression of ACTA2, SM-MHC, SM22 and CNN1 in balloon-injured carotid arteries from experimental groups. Total RNAs was isolated from carotid arteries at 48 hours after injury (n = 5). *P <0.05 versus uninjured control artery (no injury). NS: not significant. (c) Valuation of c-Fos expression in injured arteries at 2 days after balloon injury.*P<0.01 versus Injured artery from BI group. NS: not significant.

**Supplemental Figure 3**

**
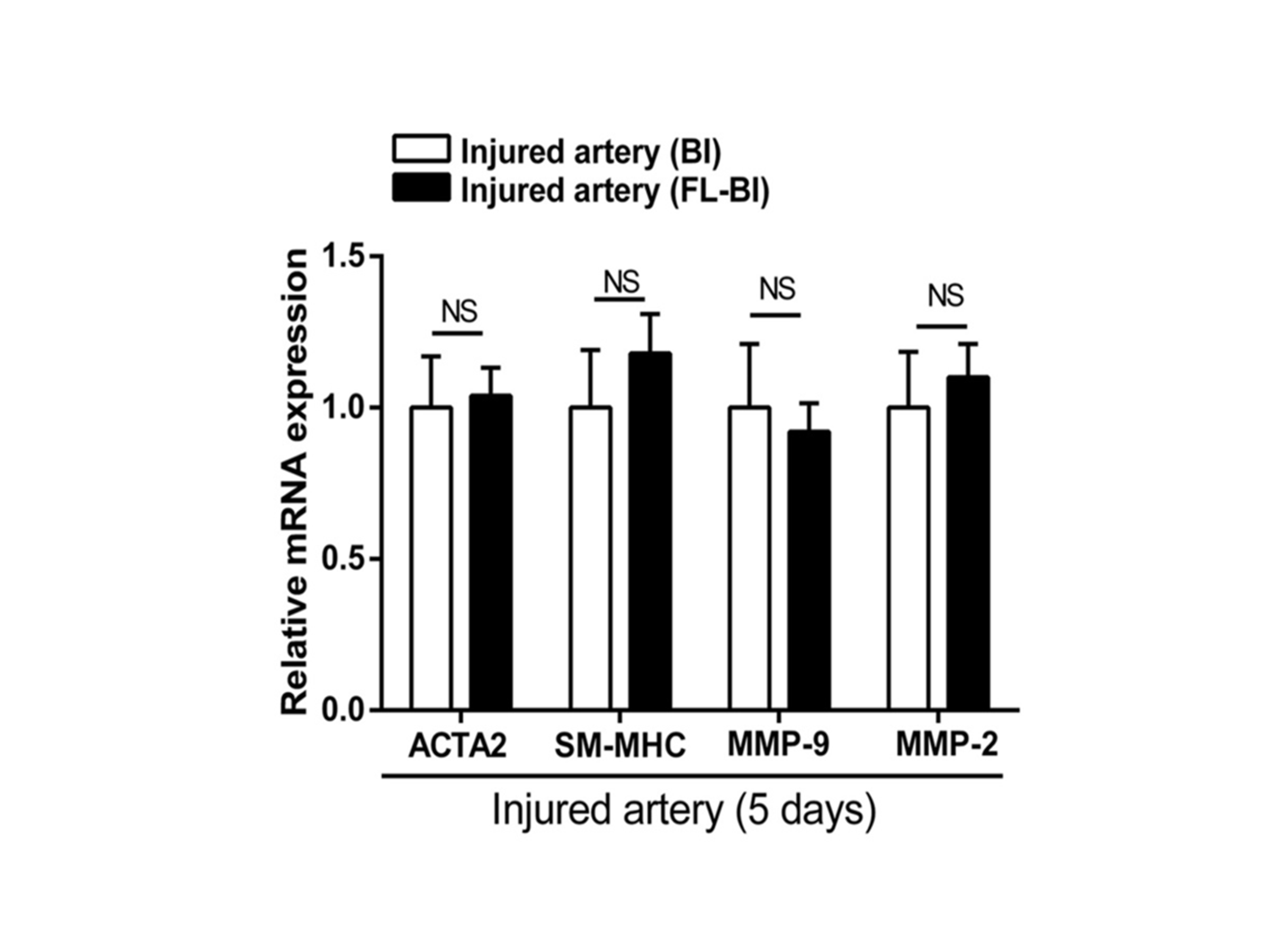
**

**Effects of hindlimb ischemia on vascular carotid remodeling at 5 days after vascular injury.** Relative expression of ACTA2, SM-MHC, MMP-9 and MMP-2 mRNA transcripts in injured artery 5 days after injury. NS: not significant; n=5.

**Supplemental Figure 4**

**
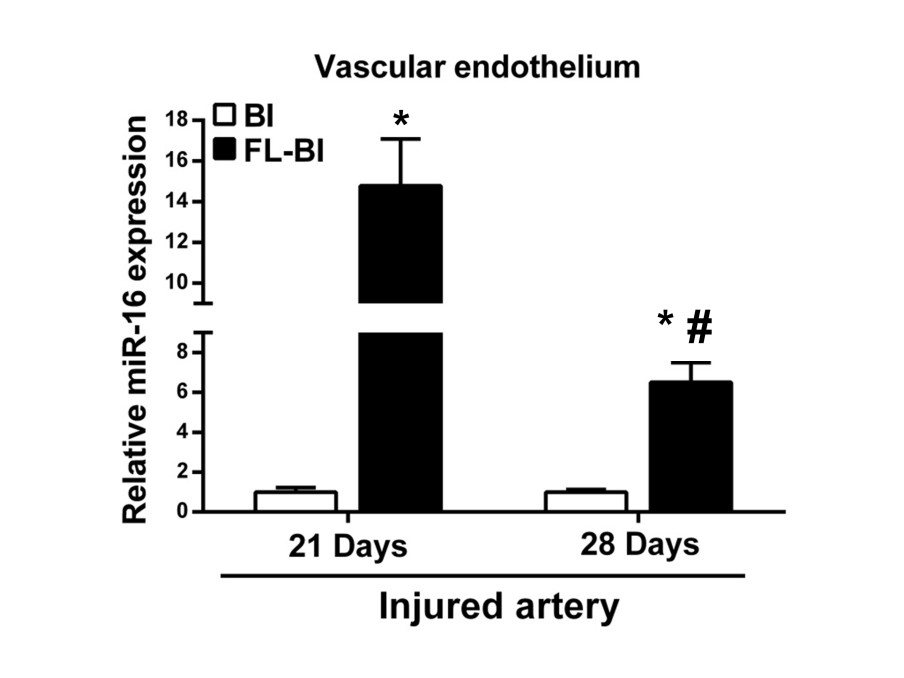
**

**Expression analysis of miR-16 in vascular endothelium .**Valuation of miR-16 expression in vascular endothelium from rat carotid arteries at 21 and 28 days after balloon injury. U6 RNA was used as the internal control to data normalization. *,P<0.05 versus Injured artery from BI group; ^#^,P<0.05 versus Injured artery from FL+BI group at 21 days. N=6

**Supplemental Figure 5**

**
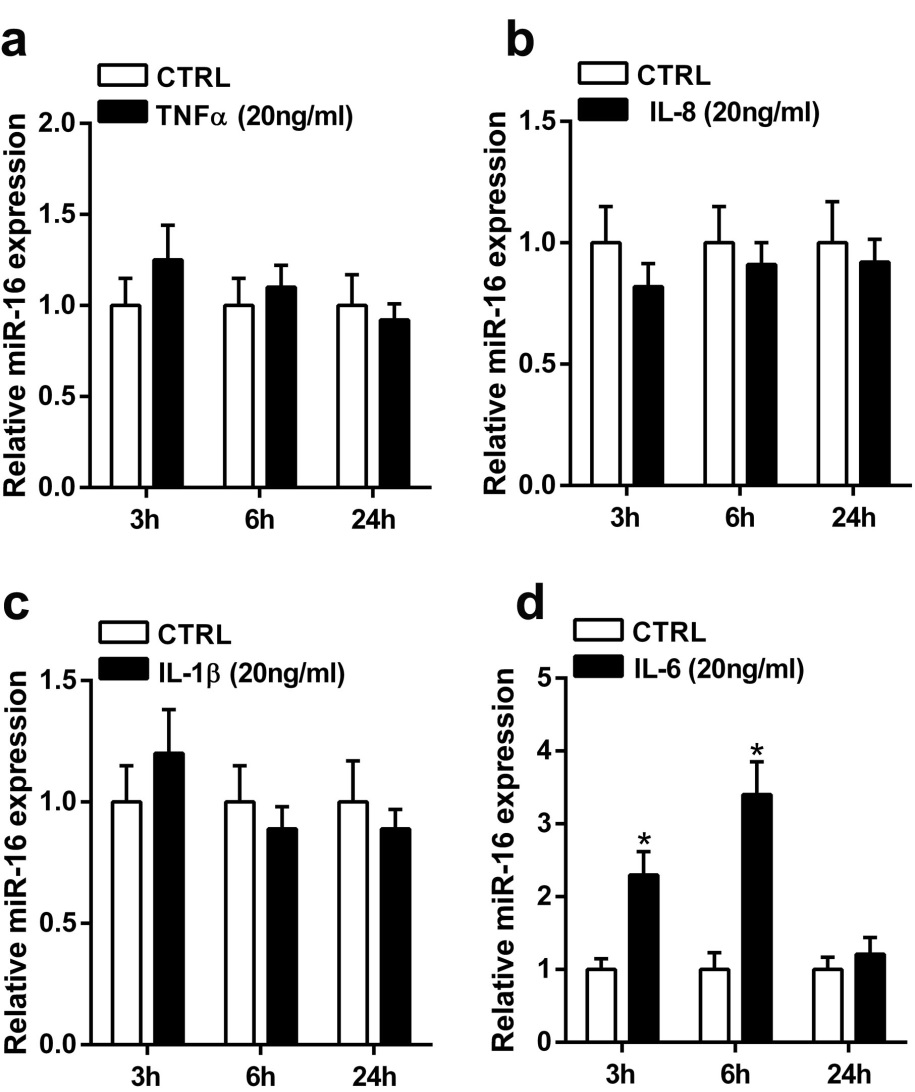
**

**Effects of pro-inflammatory cytokines on miR-16 expression in ECs.**(a-d) HUVECs were incubated with tumor necrosis factor (TNFα, 20ng/ml), interleukin-8 (IL-8, 20ng/ml), interleukin-1 β (IL-1 β, 20ng/ml) or interleukin-6 (IL-6, 20ng/ml) for 3, 6 or 24h. Real-time PCR was performed using TaqMan miRNA assays. U6 RNA was used as the internal control to data normalization. *P<0.05 vs untreated cells (CTRL). NS: not significant.

**Supplemental figure 6**

**
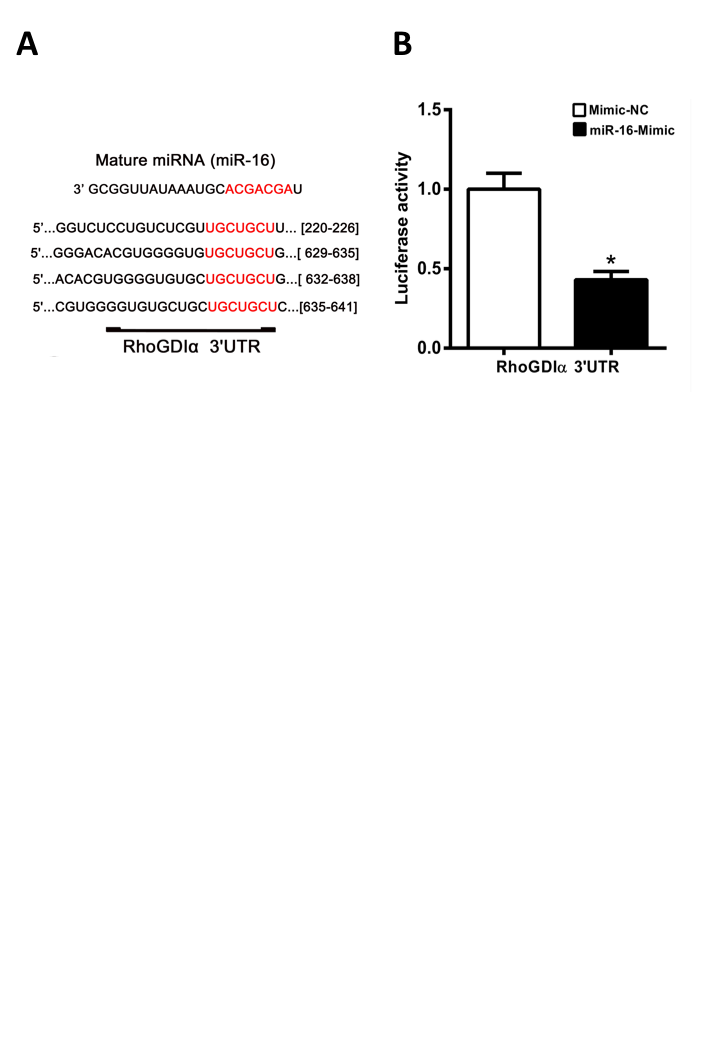
**

**RhoGDIα is a direct target of miR-16**. (a) Seed region in RhoGDIα 3’UTR recognized by miR-16. (b) 293 cells were co-transfected with indicated mimics and firefly luciferase reporter containing RhoGDIα3′UTR. After 48 hours, luciferase activity was measured. *P<0.01 versus cells transfected with Mimic-NC (n = 4).

**Supplemental Figure 7**


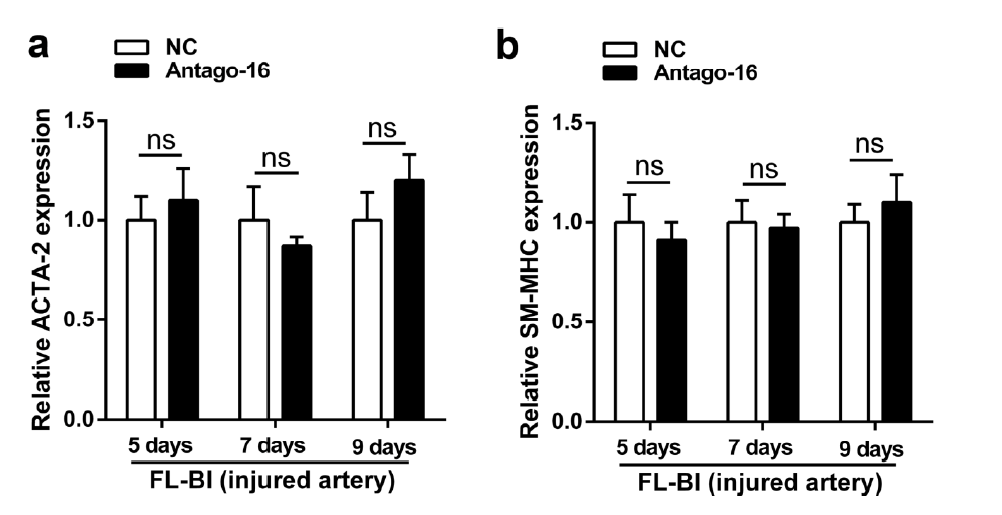


**Effect of systemic delivery of antagomiR-16 on VSMC marker expression.**(a, b) Rats were subjected to hindlimb ischemia and seven days after femoral ligation subjected to balloon injury. Rats were then randomly divided into two groups, NC group and Antago-16 group. Bar graphs represent the relative expression of ACTA-2 (a) and SM-MHC (b) mRNA transcripts in injured artery at 5, 7 and 9 days after injury. ns, not significant.

**Supplemental Figure 8**


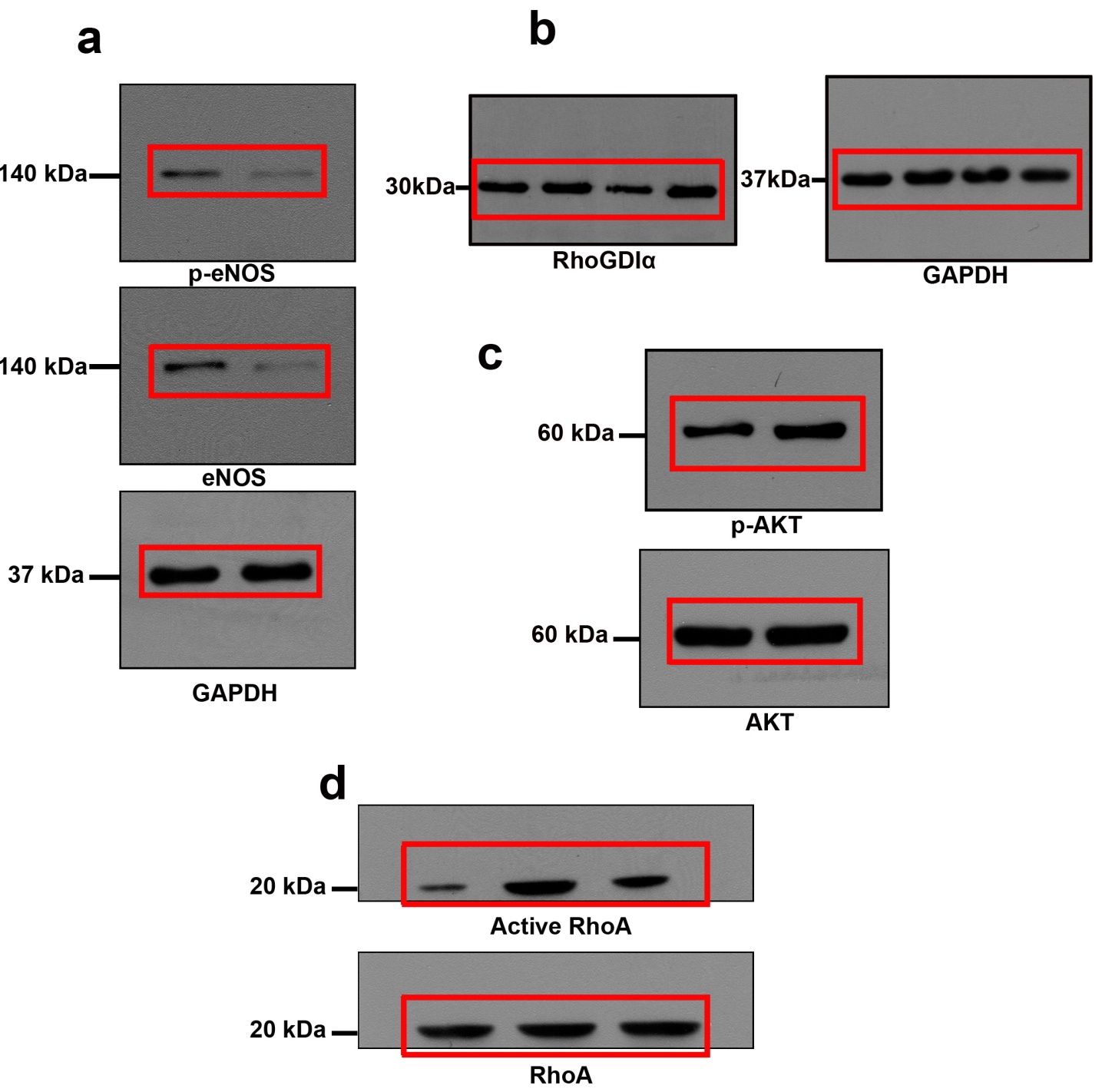


**Full-length blots of western blotting data** in Fig. 3B (a), Fig. 4C (b), Fig. 4D (c) and Fig. 4E (d). Indicated parts (red box) are shown in Fig. 3 and Fig. 4.

**Supplementary Table miRNAs included in analysis.**

**
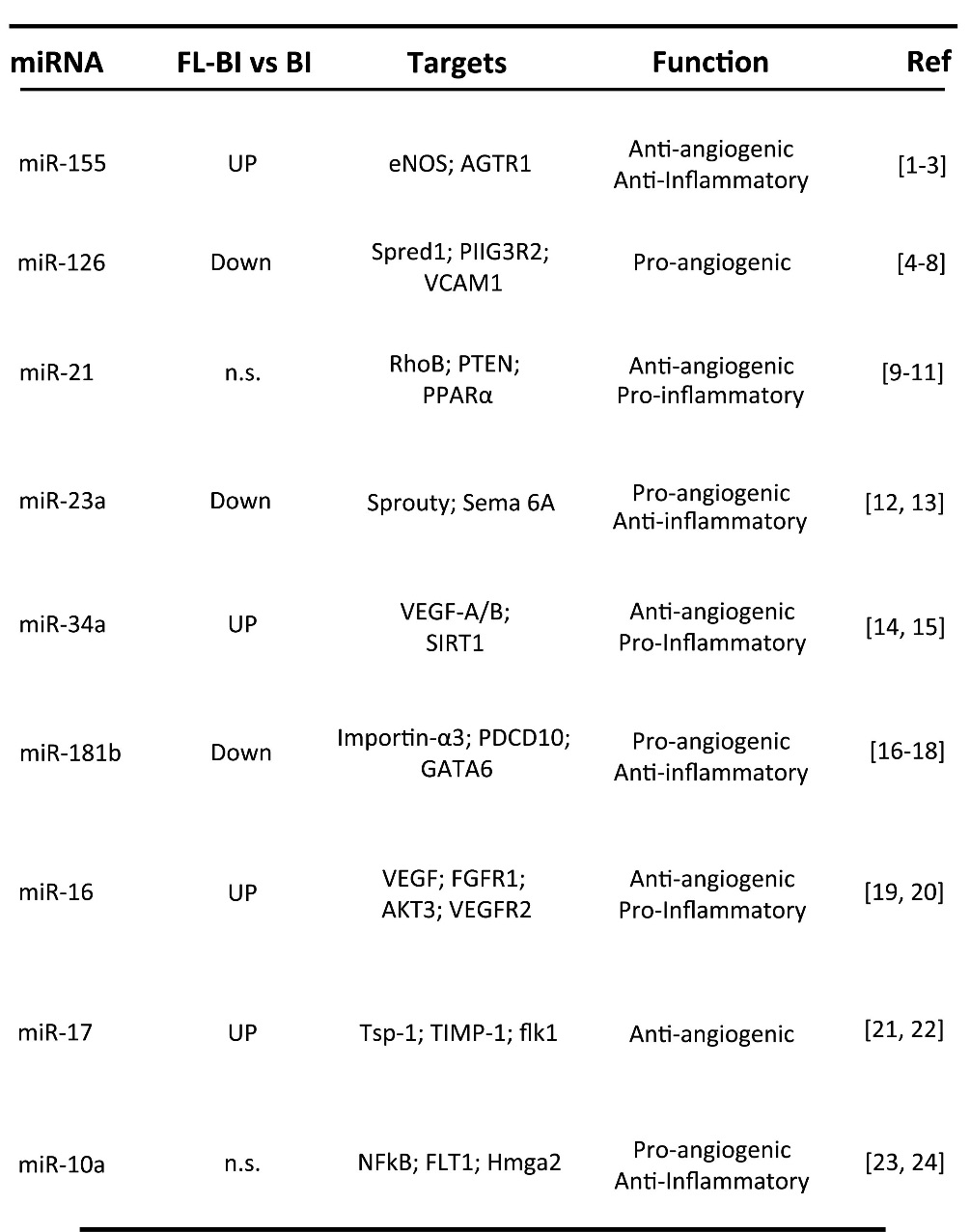
**

**Materials and Methods**

Materials and Methods are available in the online-only Data Supplement

**References**

1. Sun HX, Zeng DY, Li RT, Pang RP, Yang H, Hu YL, Zhang Q, Jiang Y, Huang LY, Tang YB, Yan GJ, Zhou JG. Essential role of microrna-155 in regulating endothelium-dependent vasorelaxation by targeting endothelial nitric oxide synthase. Hypertension. 2012;60:1407-1414.

2. Pankratz F, Bemtgen X, Zeiser R, Leonhardt F, Kreuzaler S, Hilgendorf I, Smolka C, Helbing T, Hoefer I, Esser JS, Kustermann M, Moser M, Bode C, Grundmann S. Microrna-155 exerts cell-specific antiangiogenic but proarteriogenic effects during adaptive neovascularization. Circulation. 2015;131:1575-1589.

3. Li X, Kong D, Chen H, Liu S, Hu H, Wu T, Wang J, Chen W, Ning Y, Li Y, Lu Z. Mir-155 acts as an anti-inflammatory factor in atherosclerosis-associated foam cell formation by repressing calcium-regulated heat stable protein 1. Sci Rep. 2016;6:21789.

4. Endo-Takahashi Y, Negishi Y, Nakamura A, Ukai S, Ooaku K, Oda Y, Sugimoto K, Moriyasu F, Takagi N, Suzuki R, Maruyama K, Aramaki Y. Systemic delivery of mir-126 by mirna-loaded bubble liposomes for the treatment of hindlimb ischemia. Sci Rep. 2014;4:3883.

5. Zhang Q, Kandic I, Kutryk MJ. Dysregulation of angiogenesis-related micrornas in endothelial progenitor cells from patients with coronary artery disease. Biochem Biophys Res Commun. 2011;405:42-46.

6. van Solingen C, Seghers L, Bijkerk R, Duijs JM, Roeten MK, van Oeveren-Rietdijk AM, Baelde HJ, Monge M, Vos JB, de Boer HC, Quax PH, Rabelink TJ, van Zonneveld AJ. Antagomir-mediated silencing of endothelial cell specific microrna-126 impairs ischemia-induced angiogenesis. Journal of cellular and molecular medicine. 2009;13:1577-1585.

7. Fish JE, Santoro MM, Morton SU, Yu S, Yeh RF, Wythe JD, Ivey KN, Bruneau BG, Stainier DY, Srivastava D. Mir-126 regulates angiogenic signaling and vascular integrity. Dev Cell. 2008;15:272-284.

8. Wang S, Aurora AB, Johnson BA, Qi X, McAnally J, Hill JA, Richardson JA, Bassel-Duby R, Olson EN. The endothelial-specific microrna mir-126 governs vascular integrity and angiogenesis. Dev Cell. 2008;15:261-271.

9. Sabatel C, Malvaux L, Bovy N, Deroanne C, Lambert V, Gonzalez ML, Colige A, Rakic JM, Noel A, Martial JA, Struman I. Microrna-21 exhibits antiangiogenic function by targeting rhob expression in endothelial cells. PLoS One. 2011;6:e16979.

10. Weber M, Baker MB, Moore JP, Searles CD. Mir-21 is induced in endothelial cells by shear stress and modulates apoptosis and enos activity. Biochem Biophys Res Commun. 2010;393:643-648.

11. Zhou J, Wang KC, Wu W, Subramaniam S, Shyy JY, Chiu JJ, Li JY, Chien S. Microrna-21 targets peroxisome proliferators-activated receptor-alpha in an autoregulatory loop to modulate flow-induced endothelial inflammation. Proc Natl Acad Sci U S A. 2011;108:10355-10360.

12. Zhou Q, Gallagher R, Ufret-Vincenty R, Li X, Olson EN, Wang S. Regulation of angiogenesis and choroidal neovascularization by members of microrna-23~27~24 clusters. Proc Natl Acad Sci U S A. 2011;108:8287-8292.

13. Ruan W, Xu JM, Li SB, Yuan LQ, Dai RP. Effects of down-regulation of microrna-23a on tnf-alpha-induced endothelial cell apoptosis through caspase-dependent pathways. Cardiovasc Res. 2012;93:623-632.

14. Bernardo BC, Gao XM, Winbanks CE, Boey EJ, Tham YK, Kiriazis H, Gregorevic P, Obad S, Kauppinen S, Du XJ, Lin RC, McMullen JR. Therapeutic inhibition of the mir-34 family attenuates pathological cardiac remodeling and improves heart function. Proc Natl Acad Sci U S A. 2012;109:17615-17620.

15. Fan W, Fang R, Wu X, Liu J, Feng M, Dai G, Chen G, Wu G. Shear-sensitive microrna-34a modulates flow-dependent regulation of endothelial inflammation. J Cell Sci. 2015;128:70-80.

16. Sun X, Icli B, Wara AK, Belkin N, He S, Kobzik L, Hunninghake GM, Vera MP, Registry M, Blackwell TS, Baron RM, Feinberg MW. Microrna-181b regulates nf-kappab-mediated vascular inflammation. J Clin Invest. 2012;122:1973-1990.

17. Kane NM, Howard L, Descamps B, Meloni M, McClure J, Lu R, McCahill A, Breen C, Mackenzie RM, Delles C, Mountford JC, Milligan G, Emanueli C, Baker AH. Role of micrornas 99b, 181a, and 181b in the differentiation of human embryonic stem cells to vascular endothelial cells. Stem Cells. 2012;30:643-654.

18. Xu X, Ge S, Jia R, Zhou Y, Song X, Zhang H, Fan X. Hypoxia-induced mir-181b enhances angiogenesis of retinoblastoma cells by targeting pdcd10 and gata6. Oncol Rep. 2015;33:2789-2796.

19. Spinetti G, Fortunato O, Caporali A, et al. Microrna-15a and microrna-16 impair human circulating proangiogenic cell functions and are increased in the proangiogenic cells and serum of patients with critical limb ischemia. Circ Res. 2013;112:335-346.

20. Chamorro-Jorganes A, Araldi E, Penalva LO, Sandhu D, Fernandez-Hernando C, Suarez Y. Microrna-16 and microrna-424 regulate cell-autonomous angiogenic functions in endothelial cells via targeting vascular endothelial growth factor receptor-2 and fibroblast growth factor receptor-1. Arterioscler Thromb Vasc Biol. 2011;31:2595-2606.

21. Yin R, Wang R, Guo L, Zhang W, Lu Y. Mir-17-3p inhibits angiogenesis by downregulating flk-1 in the cell growth signal pathway. J Vasc Res. 2013;50:157-166.

22. Doebele C, Bonauer A, Fischer A, Scholz A, Reiss Y, Urbich C, Hofmann WK, Zeiher AM, Dimmeler S. Members of the microrna-17-92 cluster exhibit a cell-intrinsic antiangiogenic function in endothelial cells. Blood. 2010;115:4944-4950.

23. Hassel D, Cheng P, White MP, Ivey KN, Kroll J, Augustin HG, Katus HA, Stainier DY, Srivastava D. Microrna-10 regulates the angiogenic behavior of zebrafish and human endothelial cells by promoting vascular endothelial growth factor signaling. Circ Res. 2012;111:1421-1433.

24. Fang Y, Shi C, Manduchi E, Civelek M, Davies PF. Microrna-10a regulation of proinflammatory phenotype in athero-susceptible endothelium in vivo and in vitro. Proc Natl Acad Sci U S A. 2010;107:13450-13455.
